# Risk profiling for cirrhosis and hepatocellular carcinoma in HFE hemochromatosis using mobilizable iron stores and alcohol consumption

**DOI:** 10.1038/s41598-025-99672-8

**Published:** 2025-05-08

**Authors:** Natasha D. P. Mitchell, Timothy G. St Pierre, Louise E. Ramm, Grant A. Ramm, John K. Olynyk

**Affiliations:** 1 Department of Gastroenterology, Fiona Stanley Hospital, Murdoch, WA Australia; 2 School of Physics, Mathematics, and Computing, The University of Western Australia, Perth, WA Australia; 3 Hepatic Fibrosis Group, QIMR Berghofer Medical Research Institute, Brisbane, QLD Australia; 4 Faculty of Medicine, The University of Queensland, Brisbane, QLD Australia; 5 Curtin Medical School, Curtin University, Kent St., Bentley, WA 6102 Australia

**Keywords:** Hepatocellular carcinoma (HCC), HFE hemochromatosis (HH), Hepatic iron concentration (HIC), Hepatic iron index (HII), Mobilizable iron (MobFe), Hepatology, Liver cancer, Liver cirrhosis

## Abstract

HFE hemochromatosis (HH) may cause cirrhosis and hepatocellular carcinoma (HCC). Progression to these endpoints depends on the severity of iron overload and cofactors, such as alcohol. We evaluated alcohol and iron-related risk factors in relation to cirrhosis at diagnosis and future development of HCC in a retrospective analysis of 197 HH subjects. The proportion of subjects either with cirrhosis or who developed HCC during follow-up were 29/197 (14.7%) or 10/197 (5.1%), respectively. The median (IQR) follow-up time after diagnosis was 15.2 (4.6 to 22.1) years. The median mobilizable iron stores and daily alcohol consumption (IQR) were 6.0 (3.8–11.0) g and 20 (0–40) g, respectively. An optimal logistic regression model for the odds of cirrhosis was developed by adding candidate liver insult variables (mobilizable iron, alcohol consumption, and age as a surrogate for duration of exposure) in a forward stepwise strategy using area under the receiver operating characteristic curve (AUROC) analysis and the corrected Akaike information criterion. This model demonstrated an AUROC (95% CI) of 0.966 (0.935–0.996), with sensitivity 76 (58–88)% and specificity 97 (93–99) % for prediction of cirrhosis and had a negative predictive value of 99.4 (95% CI 96.7–99.97) % for development of HCC. Thus, future risk of HCC can be assessed from mobilizable iron stores and alcohol consumption of HH subjects.

**Supplementary Information:**

The online version contains supplementary material available at 10.1038/s41598-025-99672-8.

## Introduction

HFE hemochromatosis (HH) is an autosomal recessive inherited disorder of iron overload which most commonly is caused by a homozygous C282Y mutation in the *HFE* gene^1^. This genetic defect affects approximately 1 in 200 individuals of European origin and impairs the regulation of hepcidin, the principal negative iron-regulatory hormone, resulting in clinically significant iron overload in up to 40% of C282Y homozygous individuals^2–4^.

One of the most serious sequelae of iron overload is cirrhosis, which may occur in up to 9% of men and 2% of women with HFE hemochromatosis^4–6^. Cirrhosis increases the future lifetime risk of hepatocellular carcinoma (HCC) up to 12-fold, especially in men^7^. The risk of developing cirrhosis is associated with the degree and duration of iron overload^8–10^. Iron overload can be assessed by estimating mobilizable iron from phlebotomy, the mainstay treatment for iron overload in hemochromatosis^11^. The progression to cirrhosis and HCC varies beyond that which can be attributed to iron overload alone^12^. Alcohol has been considered a significant risk factor in the development of cirrhosis in HH, increasing the risk 9-times in patients drinking more than 60 g of alcohol per day^13^. This is accompanied by a 2.3-fold increased relative risk of HCC, especially in those drinking more than 80 g of alcohol per day^14^. Whilst liver fibrosis and cirrhosis have, in the past, been diagnosed following liver biopsy, clinicians are now more commonly using validated non-invasive methods such as blood biomarker panels, including the fibrosis-4 or aspartate aminotransferase- to- platelet ratio index, or elastography^15,16^.

Given the potential synergism between iron overload and alcohol consumption, we explored the interaction between mobilizable iron stores and alcohol consumption as risk factors for future development of cirrhosis and HCC in a retrospective analysis of 197 well-characterized C282Y homozygous subjects who had undergone liver biopsy at diagnosis between 1974 and 2010.

## Results

### Baseline characteristics

**Fig. 1:**
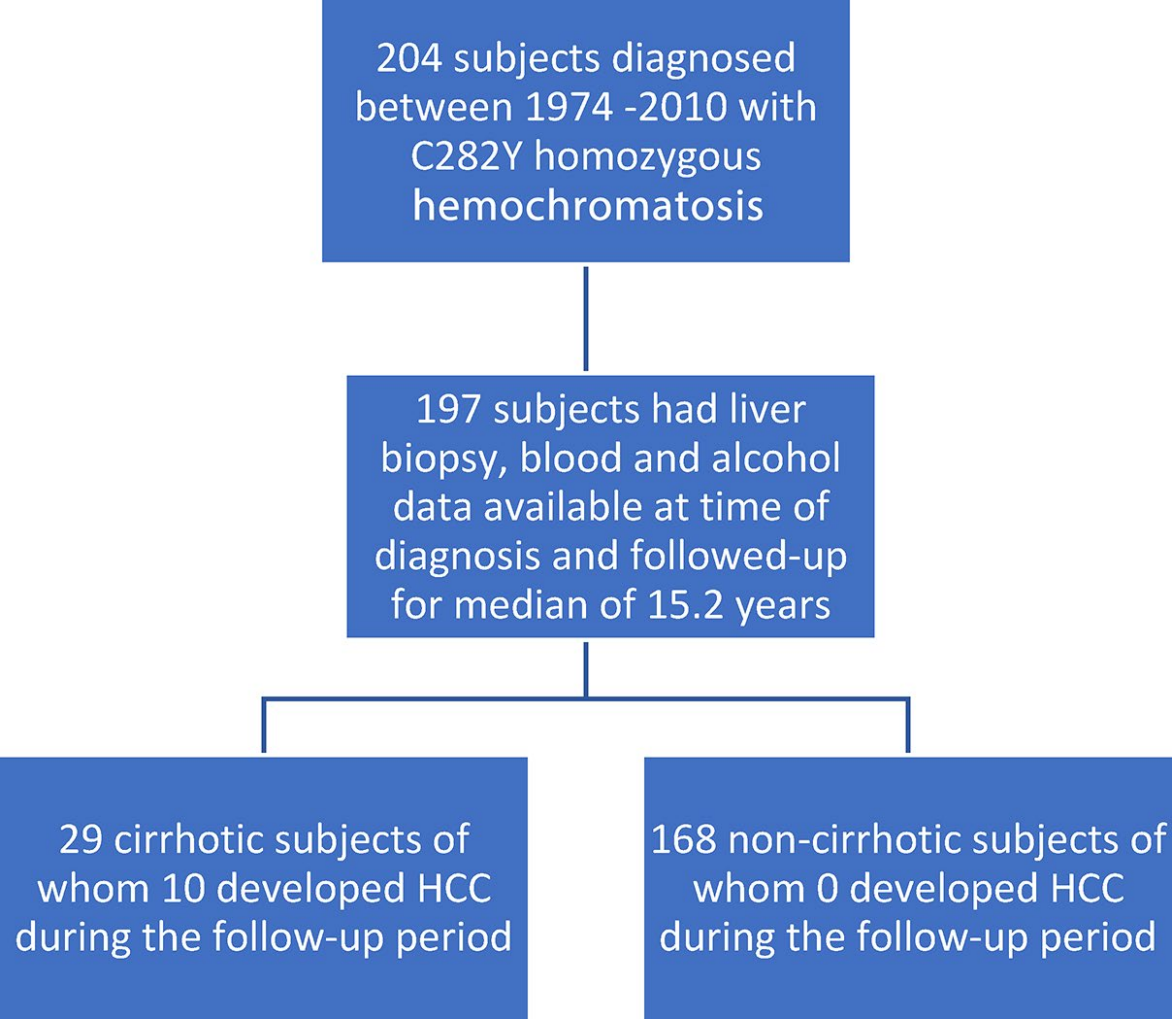
Flow chart of subjects included in the study. 204 subjects diagnosed with HH between 1974 and 2010, of those 197 had completed clinical information and they were followed up for a median of 15.2 years. 29 patients had liver biopsy proven cirrhosis, of those 10 developed HCC during the follow-up period. 168 did not have liver biopsy proven cirrhosis, and none of these patients developed HCC during the follow-up period.

Figure 1, shows a flow chart of the 204 HH subjects identified in the database, 7 had no records of alcohol intake leaving 197 patients with full records of age at diagnosis, liver histology, hepatic iron concentration (HIC), hepatic iron index (HII), mobilized iron, and alcohol (EtOH) intake; 29 of these had cirrhosis. Table 1 shows the baseline characteristics of the study subjects. Steatosis data were available for 174 or the 197 subjects [107/174 (61%) grade 0; 45/174 (26%) grade 1; 16/174 (9%) grade 2; 6/174 (3%) grade 3]. Body mass index (BMI) was similar in subjects with or without cirrhosis or HCC. Duration of follow-up was available for 153/197 subjects with the median (IQR) follow-up period being 15.2 (4.6 to 22.1) years. The available median follow-up periods for subjects who did (n = 10) or did not (n = 143) develop HCC were 14.4 and 15.2 years respectively (*p* = 0.84, Mann–Whitney test). Six of 29 subjects (21%) who were diagnosed with cirrhosis died during study follow-up compared with 6 of 168 noncirrhotic subjects (3.6%, *P* = 0.0030, Fisher’s exact test). Of the 6 cirrhotic subjects who died, 5 developed HCC.

**Table 1:** Patient characteristics.

Characteristics	All patients	No cirrhosis	Cirrhosis	*p*-value	Cirrhosis without development of HCC	Cirrhosis with development of HCC	*p*-value
N	197	168	29		19	10	
Male, N (%)	133 (68%)	106 (63%)	27 (93%)	0.001	17 (89%)	10 (100%)	n.s
Age (y)	43.8 (SD 13.7)	42.5 (SD 13.7)	51.4 (SD 10.6)	0.0012	51.0 (SD 9.9)	52.1 (SD 12.4)	n.s
Serum ferritin (μg/L)	973 (498–1853) ^1^	812 (461–1291) ^2^	3360 (2432 -4255)	< 0.0001	4040 (2290–4220)	2795 (2463–3853)	n.s
TSat (%)	85 (73–97) ^3^	84 (71–96) ^4^	90 (78–98) ^5^	0.048	84 (78 -96) ^6^	98 (90–100)	0.03
Mobilizable Fe (g)	6.0 (3.8–11.0)	5.4 (3.5–7.7)	18.0 (13.5–25.0)	< 0.0001	17.0 (13.0–25.0)	24.5 (13.6–26.8)	n.s
Alcohol intake (g/d)	20 (0–40)	10 (0–30)	60 (20–100)	< 0.0001	40 (20–80)	100 (17.5–112.5)	n.s
HIC (μmol Fe/g dw.)	154 (103–258)	144 (88–216)	324 (201–386)	< 0.0001	335 (201–386)	276 (163–395)	n.s
HII (μmol Fe/(g dw. y))	3.9 (2.5–6.0)	3.6 (2.1–5.6)	5.7 (4.1–8.2)	0.0001	6.2 (4.9–8.0)	4.9 (3.4–9.0)	n.s
Steatosis	67/174 (39%)	56/149 (38%)	11/25 (44%)	n.s	9/15 (60%)	2/10 (20%)	n.s
BMI (kg/m^2^)	26.5 (SD 4.5)^7^	26.4 (SD 4.6)^7^	26.7 (SD 4.2)^7^	n.s	27.9 (SD 4.0)^7^	24.1 (SD 3.4)^1^	n.s

Age—age at time of biopsy; TSat—transferrin saturation; dw—dry weight; HIC—hepatic iron concentration measured from biopsy; HII—hepatic iron index; Steatosis—presence of steatosis on biopsy (note that steatosis information was available for only 174 of the patients); BMI—body mass index. Continuous variables are presented as the mean and standard deviation if normally distributed or as the median and interquartile range otherwise. Distributions of variables are compared with the t-test, if normally distributed or with the Mann–Whitney test otherwise. Association of categorical variables was tested with Fisher’s Exact test. ^1^ Serum ferritin available for 193/197 patients; ^2^ serum ferritin available for 164/168 patients; ^3^ transferrin saturation available for 191/197 patients; ^4^ transferrin saturation available for 163/168 patients; ^5^ transferrin saturation available for 28/29 patients; ^6^ transferrin saturation was available for 18/19 patients; ^7^ BMI (body mass index) data were available for 121/197 patients (105/168 without and 16/29 with cirrhosis; 11/19 cirrhosis without development of HCC and 5/10 cirrhosis with development of HCC).

Table 2 shows the strength and statistical significance of association of each of the predictor variables with the diagnosis of cirrhosis in terms of both the area under the receiver operating characteristic curve (AUROC) and the Mann–Whitney p-value. Mobilizable iron (*MobFe*) had the strongest association with cirrhosis and hence was selected as the key variable to be included in all tested logistic regression models for the odds of cirrhosis (Table 2).

**Table 2:** Strength of association of each individual predictor variable with diagnosis of cirrhosis.

Predictor variable (units)	AUROC [95% CI]	P-value
*MobFe* (g)	0.96 [0.93–0.99]	< 0.0001
*HIC* (μmol/g dw)	0.82 [0.74–0.90]	< 0.0001
*EtOH* (g per day)	0.76 [0.65–0.86]	< 0.0001
*HII* (μmol Fe/(g dw. year))	0.72 [0.63–0.81]	0.0001
*Age* (years)	0.70 [0.61–0.79]	0.0005

AUROC—area under receiver operating characteristic curve; 95% CI—95% confidence interval; p-value from Mann–Whitney test comparing variable values between cirrhotic and non-cirrhotic patients; *MobFe*—mobilized iron; *HIC*—hepatic iron concentration from biopsy; dw—dry weight; *EtOH*—alcohol consumption per day; *HII*—hepatic iron index; *Age*—age at diagnosis.

The correlation matrix for the 5 candidate predictor variables is shown in Table 3. *HIC* and *HII* had the strongest correlations with *MobFe* and hence were eliminated from further consideration as candidate predictors since their inclusion would increase uncertainty on the coefficients of any derived model^17^. The remaining candidate predictor variables *MobFe*, *EtOH*, and *Age* had low correlations with each other (Table 3) and hence were retained as candidate predictor variables.

**Table 3:** Spearman rank order correlation coefficient matrix showing the strength of correlation between the candidate predictor variables.

	HIC	MobFe	Age	EtOH	HII
HIC	1	0.73	0.23	0.21	0.85
MobFe	0.73	1	0.24	0.36	0.58
Age	0.23	0.24	1	0.13*	− 0.26
EtOH	0.21	0.36	0.13*	1	0.13*
HII	0.85	0.58	− 0.26	0.13*	1

All correlations had *p*-values < 0.004 except those marked with * which had *p*-values > 0.05.

The supplementary table shows the forward stepwise approach used to build the logistic regression model for the odds of cirrhosis starting with the simplest model, Model 1. Both the AUROC and corrected Akaike information criterion indicate sequential improvement of the models up to Model 3. While Model 4 has a better AUROC than Model 3, the corrected Akaike information criterion indicates that it is less likely to be the correct model and not all of the model coefficients are significantly different from zero. Model 5 includes *Age* as a predictor, and compared with Model 3 has superior AUROC, with all coefficients being significantly different from zero, with the corrected Akaike information criterion indicating that it is the model that is more likely correct. However, Model 5 does not satisfy the criterion that there are more than 10 cirrhosis cases per predictor variable and hence was rejected on the grounds that fewer than 10 events per variable can increase the risk of false positive findings and reduce model performance^17,18^. All other possible models fail this criterion as well, resulting in Model 3 being identified as the best model for the data.

### Model 3 has the following form

$${\text{Ln}}\left( {odds} \right) = - {6}.{472} + 0.{2833} \times MobFe + 0.00{3}0{6}0 \times MobFe \times EtOH$$

where *odds* is the predicted odds of a patient having cirrhosis at diagnosis.

**Fig. 2:**
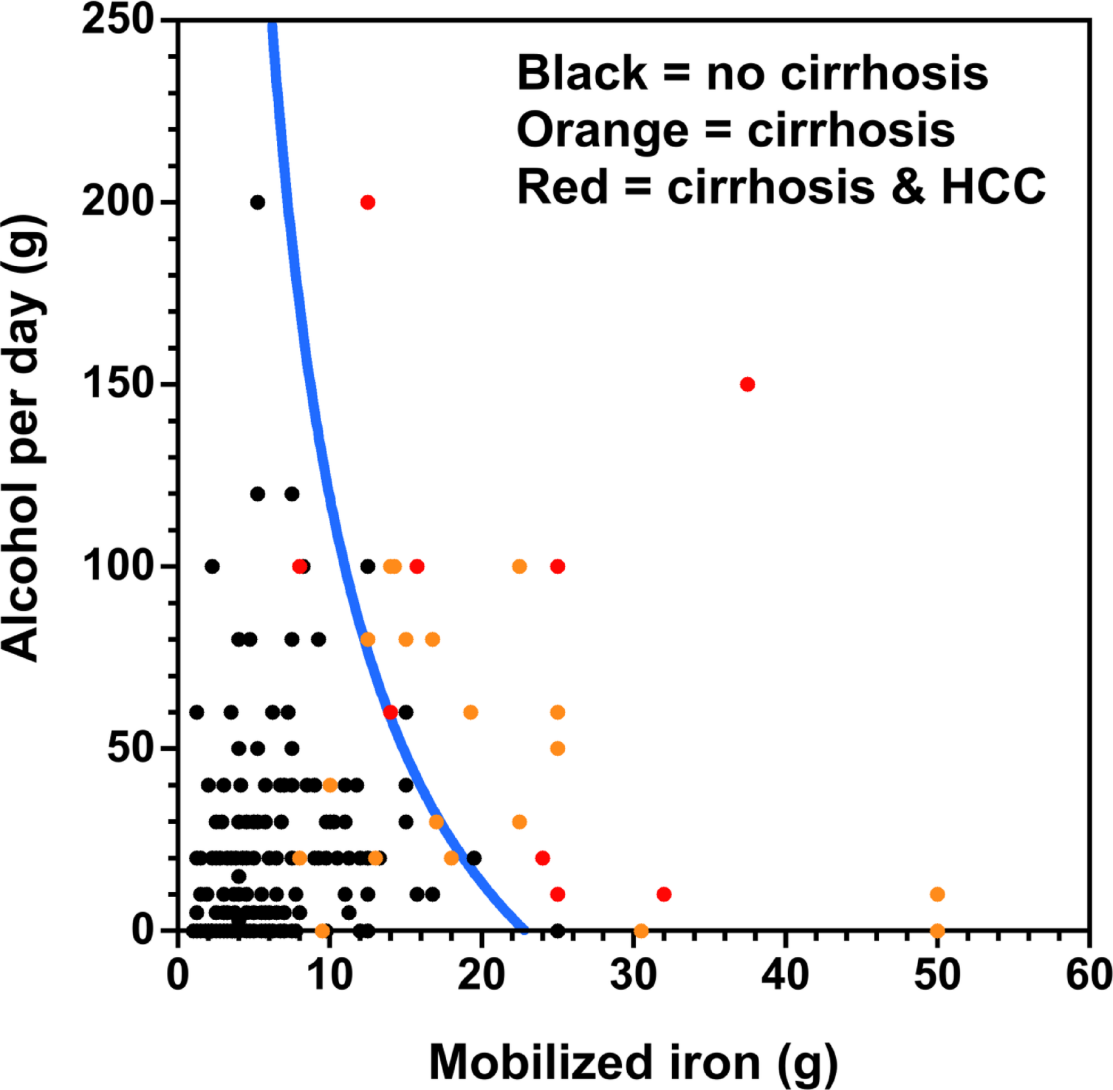
Scatterplot of alcohol consumed per day against mobilized iron. The blue line indicates the loci of points where the predicted odds ratio for cirrhosis is 1.0. Note that two of the HCC datapoints overlap. All patients with HCC had cirrhosis.

Figure 2 shows a scatterplot of *EtOH* against *MobFe* for the 197 subjects. The blue line represents the loci of points where the odds ratio predicted by Model 3 is 1.0 i.e. a predicted 50% likelihood of cirrhosis. The selection of Model 3 over Model 4 indicates that any independent impact of alcohol in these patients is not of sufficient magnitude to warrant inclusion in the modelling. The curved nature of the blue line is a result of the inclusion of the interaction term between *MobFe* and *EtOH* in the model indicating that the odds of cirrhosis is not due simply to an addition of the two insults but is a result of an interaction between alcohol and iron.

**Fig. 3:**
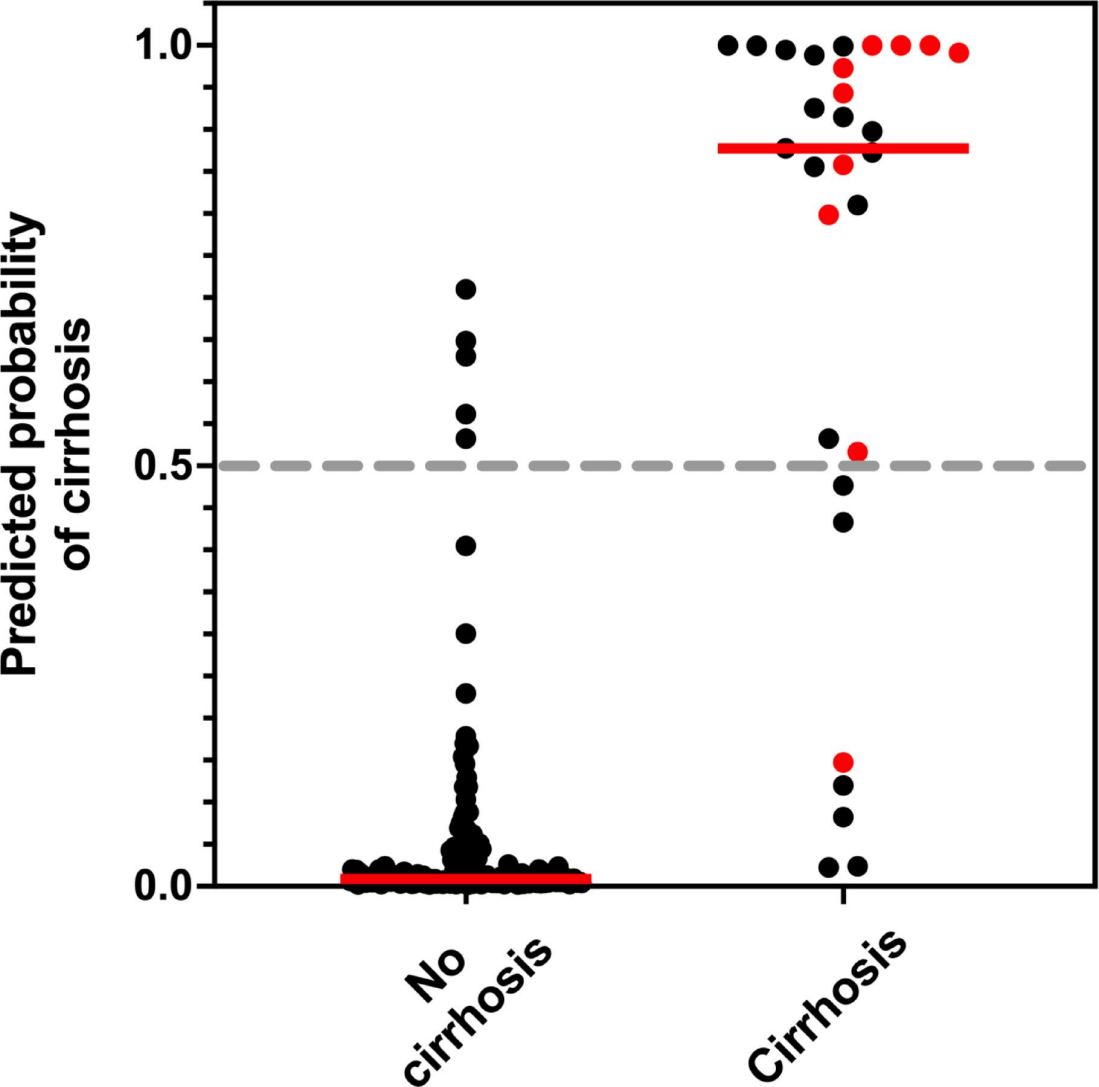
Probabilities of cirrhosis estimated by Model 3 for patients diagnosed without (N = 168) and with (N = 29) cirrhosis. The solid red lines represent the median predicted probabilities. Red datapoints indicate patients diagnosed with HCC.

Figure 3 shows the probabilities of cirrhosis estimated by Model 3 for subjects who either developed or did not develop cirrhosis. Model 3 has a sensitivity [95% CI] of 76 [58–88]%, a specificity of 97 [93–99]%, a positive predictive value of 81 [62–92]%, and a negative predictive value of 96 [92–98]% for predicting cirrhosis using the odds ratio cutoff of 1.0 (i.e. probability cutoff of 0.5).

Given that cirrhosis has been observed to increase the future lifetime risk of HCC up to 12-fold, especially in men (Atkins et al., 2020), the diagnostic performance of Model 3 for predicting the development of HCC was tested. Using the odds cutoff of 1.0, the negative predictive value was the most accurately determined diagnostic performance parameter being 99.4 [96.7–99.97] %. The positive predictive value was 33 [19–52] %; sensitivity was 90 [60–99] %; specificity was 90 [85–94] %; and AUROC of 95.5 [92.1–99.1] %. Overall, 9 of 10 cases of HCC which occurred in the cohort of 197 subjects were correctly identified by Model 3 as being at increased risk of HCC at the time of original diagnosis, many years prior to development of the malignancy (Fig. 3).

## Discussion

This study of 197 well-characterized HH subjects who had undergone liver biopsy as a standard-of-care has enabled the development of a logistic regression model that enables risk profiling for cirrhosis and future development of HCC using mobilizable iron stores and alcohol consumption. The strengths of the model are a high specificity and negative predictive value for prediction of cirrhosis and a high negative predictive value regarding prediction of HCC. Further validation in an independent cohort would facilitate broader uptake and clinical use to risk stratify those most at risk of HCC and requiring surveillance.

Interestingly, mobilizable iron had a stronger univariate relationship with cirrhosis compared with HIC measured from biopsy despite HIC reflecting iron in direct contact with the liver. A possible explanation for this difference may be due to the large measurement uncertainties on HIC measured by biopsy owing to the small sample size of liver material classically used for biochemical measurement, the spatial heterogeneity of iron concentration in the liver, and increased variability in the presence of fibrosis and cirrhosis^19,20^. These factors may degrade the association of HIC with cirrhosis, leaving mobilizable iron as the measure with the strongest univariate relationship with cirrhosis. Future studies with more precise MRI measures of HIC such as R2-MRI may resolve this question and lead to more accurate models for prediction of cirrhosis^21,22^.

Prior studies have shown the association between HH, alcohol intake and the increased risk of cirrhosis. However, the amount of alcohol in these studies varies from 20 to 80 g per day^13,23–25^. A key observation from our modelling is that there is an interaction between mobilizable iron and alcohol intake with regard to the odds of cirrhosis, suggesting that the combined insult to the liver from iron and alcohol is more than simply the sum of independent insults. The mechanism behind this has been explored in various animal studies. Mice fed alcohol and iron have a three-fold increase in lipid peroxidation and a two-fold increase in oxidative DNA damage compared to mice fed either alcohol or iron alone^26^. Gao et al. (2017) show that co-administration of low alcohol and iron results in significant liver steatosis and necrosis, whereas the groups given either low alcohol or low iron showed minimal evidence of liver injury^27^. Additionally, alcohol is thought to worsen iron overload via downregulation of hepcidin and increased absorption through the gastrointestinal tract^28,29^.

Bardou-Jacquet et al., have shown that excessive alcohol, more than 21 drinks per week in men and more than 14 drinks per week in women, is associated with a three-times increased risk of HCC in HH subjects with liver biopsy-proven cirrhosis^30^. Conversely, liver iron overload occurring in carriers of the C282Y mutation is associated with HCC in subjects with alcoholic cirrhosis^31^. In our study, all of the subjects with HCC had cirrhosis and six of them had alcohol intake more than the recommended limit of 40 g per day^32^. It is likely that multiple insults on a vulnerable HH-cirrhotic liver increases the risk for HCC. Bardou-Jacquet et al., highlight the importance of reducing risk factors, such as alcohol, to enhance the benefit of iron removal from venesection to improve fibrosis regression, thus reducing the risk of HCC^30^.

The model developed here provides a potential method of risk assessment for cirrhosis and HCC, accessible in an outpatient clinic setting. Alongside other non-invasive risk assessments, this model could assist clinicians in identifying those individuals at greatest or lowest risk of cirrhosis and HCC, facilitating rational clinical care decision-making, especially with regard to recommendations for liver biopsy or HCC surveillance^15^. A number of non-invasive methods have recently been described for assessing hepatic fibrosis and hepatic iron concentration in subjects with HH. Blood-based biomarker panels including an aspartate aminotransferase-to- platelet- ratio index (APRI) greater than 0.44 and a fibrosis-4 (FIB-4) greater than 1.1 exhibit greater than 86% diagnostic accuracy for advanced hepatic fibrosis or cirrhosis in HH^15^. The MRI R2 method has also been shown to be a non-invasive method for measurement of hepatic iron concentration that is more accurate than biopsy^21,22^. Current guidelines recommend 6-monthly surveillance using imaging (ultrasound, CT scan or MRI) for HCC in those with cirrhosis or otherwise deemed to be high risk for HCC^1^. Unfortunately, only 40% of individuals recommended to undergo this surveillance actually participate^33^. We found that the risk of death is significantly increased in subjects who are diagnosed with cirrhosis, primarily due to development of HCC. Our model could help risk-stratify individuals most in need of surveillance, thus helping to potentially improve clinical outcomes from surveillance in HH.

There are some potential limitations of our study. We relied on liver biopsy for diagnosis of cirrhosis and had a limited follow-up period for those subjects diagnosed with HCC towards the end of the study. The risk of HCC depends on the duration of cirrhosis^34^. Therefore, we may have missed possible HCC diagnoses in subjects who were diagnosed in the later part of the study time-period when follow-up might have been shorter. A longer period of follow-up may have resulted in more cases being identified. Self-reported alcohol intake may be inaccurate and thus could be a cause for bias^35^. Additionally, further testing of the model in an independent cohort of individuals with HH using non-invasive assessments for hepatic iron concentration and fibrosis is recommended to confirm the applicability of the model in more contemporary times.

In conclusion, we have produced a model using mobilizable iron and alcohol intake to aid in assessing the risk of cirrhosis and future HCC in HH subjects. Our findings provide further confirmation for an interaction between alcohol and iron overload significantly influencing clinical outcomes in HH. Further validation of this model is recommended to optimise its use as a risk analysis tool for clinical decision making.

## Methods

In a recent study of the relationship between extrahepatic iron loading and liver biopsy-proven fibrosis stage^36^, 204 people diagnosed between 1974 and 2010 with HFE haemochromatosis (138 men and 66 women) were identified from the QIMR Berghofer Medical Research Institute Haemochromatosis database. These individuals all were clinically well characterized and had available information derived from serum iron biochemistry, histological staging of liver fibrosis, steatosis grade, hepatic iron concentration (HIC, at the time of diagnosis), and mobilizable iron stores following phlebotomy treatment^36^. Exclusion criteria included age less than 16 years or other forms of chronic liver disease, including chronic viral hepatitis, immune-mediated liver and known metabolic liver diseases. All subjects were untreated at the time of their study inclusion. Detailed alcohol consumption records were available in 197 of 204 subjects at the time of diagnosis, and these comprised the subjects for the current study.

Alcohol consumption was assessed in grams of ethanol equivalent per day. The National Health and Medical Research Council of Australia definition for alcohol consumption (1 standard drink contains 10 g of alcohol) was used to record alcohol consumption.

Mobilizable iron stores were calculated by multiplying the number of units of blood removed by weekly phlebotomy to reduce the serum ferritin to 50–100 μg/L by 250 mg (the quantity of iron removed with each unit of blood). The hepatic iron concentration HIC was measured by atomic absorption spectrophotometry on fresh biopsy specimens as previously described^37^.

Liver biopsy-based fibrosis staging was conducted according to the Scheuer classification by histopathologists with expertise in hemochromatosis: F0–no fibrosis, F1–mild fibrosis with enlarged portal tracts, F2–moderate periportal and portal-portal septa but intact architecture, F3–severe fibrosis with architectural distortion; and F4–cirrhosis with architectural distortion^38^. Steatosis was recorded as normal, mild, moderate, or severe (represented as grades 0 to 3)^38^.

The study was approved by the Human Research Ethics Committees of the Royal Brisbane and Women’s Hospital and the QIMR Berghofer Medical Research Institute, Brisbane, Australia and informed written consent was obtained at the time of entry into the study. This study is in compliance with the Australian Code for the Responsible Conduct of Research, 2018.

### Statistical analysis

In order to elucidate the key factors leading to cirrhosis and hence increased risk of HCC, multiple logistic regression models for the odds of the presence of cirrhosis at diagnosis were developed using the following measures of insult as variables: mobilized iron (*MobFe*) in g, hepatic iron concentration from biopsy (*HIC*) in μmol/g dry weight, alcohol consumption (*EtOH*) in g per day, and hepatic iron index (*HII*) in μmol Fe/g divided by age (yrs) at diagnosis. Age at diagnosis (*Age*) in years was used as a surrogate measure of duration of exposure to iron overload and injury. The strength of each individual variable for predicting cirrhosis was tested using the area under the receiver operating characteristic curve with 95% confidence intervals being calculated by the method of Hanley and McNeil^39^. The correlations between each pair of variables were evaluated using Spearman’s correlation coefficient. The correlation coefficients together with the area under the receiver operating characteristic curve (AUROCs) were used to identify the best potential models for the data. Highly correlated variables were deemed ineligible for the models. The supplementary table shows the forward stepwise approach that was taken to selecting variables for the models, variables and interaction terms being added one by one until no further improvement of the model was observed.

Models were compared using the corrected Akaike’s information criterion^40^. The number of variables in eligible models was limited such that both the number of cases of cirrhosis and the number of cases of cirrhosis-free patients were required to be greater than 10 per predictor variable to avoid over-fitting^18^.

GraphPad Prism version 10.3.1 was used for statistical tests, logistic regression modelling, and the plotting of Fig. 3. KaleidaGraph version 5.0.4 was used to produce Fig. 2.

## Electronic supplementary material

Below is the link to the electronic supplementary material.

Supplementary Material 1

Supplementary Material 2

## Data Availability

The data supporting the findings of this study are not publicly available due to sensitivity concerns. For inquiries regarding access, please contact the corresponding author.
