# Supplementary material for: Risk profiling for cirrhosis and hepatocellular carcinoma in HFE hemochromatosis using mobilizable iron stores and alcohol consumption
Source: Sci Rep. 2025 May 8;15:16011. doi: 10.1038/s41598-025-99672-8 (PMC12062215; doi:10.1038/s41598-025-99672-8)
Supplement: Supplementary file 2 — Supplementary Material 2 [file 41598_2025_99672_MOESM2_ESM.docx]

**Supplementary Information**

| **Model** | **Model description** | **AUROC** | **95% CI on AUROC** | **p-value** | **All coefficients significant?** | **Comparison** | **Akaike IC probability (%)** | **Cirrhosis cases per predictor variable** |
| --- | --- | --- | --- | --- | --- | --- | --- | --- |
| Model 1 | β0+β1**MobFe* | 0.956 | 0.926 to 0.986 | <0.0001 | Y |  |  | 29 |
| Model 2 | β0+β1**MobFe*+β2**EtOH* | 0.962 | 0.930 to 0.994 | <0.0001 | Y | Model 2 vs 1 | 99.6 | 14.5 |
| Model 3 | β0+β1**MobFe*+β2**EtOH***MobFe* | 0.966 | 0.935 to 0.996 | <0.0001 | Y | Model 3 vs 2 | 92.3 | 14.5 |
|  |  |  |  |  |  | Model 3 vs 1 | 99.9 |  |
| Model 4 | β0+β1**MobFe*+β2**EtOH*+β3**EtOH***MobFe* | 0.967 | 0.939 to 0.996 | <0.0001 | N | Model 4 vs 3 | 30.8 | 14.5 |
| Model 5 | β0+β1**MobFe*+β2**EtOH***MobFe*+β3**Age* | 0.979 | 0.959 to 0.999 | <0.0001 | Y | Model 5 vs 3 | 98.4 | 9.7 |

Supplementary Table. The table shows the forward stepwise approach to increasing the complexity of the cirrhosis prediction models with Model 1 being the most simple model. β0, β1, β2, β3 are the model coefficients derived from the multiple logistic regression; AUROC is the area under the receiver operating characteristic curve; p-value is from the Mann-Whitney test comparing cirrhotic with non-cirrhotic patients with the model; coefficients are considered to be significant if their p-value is <0.05; the probability that the given model is more likely than the comparator model is derived from the corrected Akaike Information Criterion.
